# Supplementary material for: Cryo-EM of mammalian PA28αβ-iCP immunoproteasome reveals a distinct mechanism of proteasome activation by PA28αβ
Source: Nat Commun. 2021 Feb 2;12:739. doi: 10.1038/s41467-021-21028-3 (PMC7854634; doi:10.1038/s41467-021-21028-3)
Supplement: Supplementary file 1 — Supplementary Information [file 41467_2021_21028_MOESM1_ESM.pdf]

Supplementary Information for:

Cryo-EM of mammalian PA28αβ-iCP immunoproteasome reveals a distinct mechanism of proteasome activation by PA28αβ

Jinhuan Chen[1,#], Yifan Wang[1,2,#], Cong Xu[1,2,#], Kaijian Chen[1,2], Qiaoyu Zhao[1,2], Shutian Wang[1,2], Yue Yin[3], Chao Peng[3,*], Zhanyu Ding[1,*], Yao Cong[1,2,4,5*]

[1] State Key Laboratory of Molecular Biology, National Center for Protein Science Shanghai, Shanghai Institute of Biochemistry and Cell Biology, Center for Excellence in Molecular Cell Science, Chinese Academy of Sciences, Shanghai 200031, China

[2] University of Chinese Academy of Sciences, Beijing 100049, China

[3] National Facility for Protein Science in Shanghai, Zhangjiang Lab, Shanghai Advanced Research Institute, CAS, Shanghai, 201210, China

[4] Shanghai Science Research Center, CAS, Shanghai, China 201210

[5] Lead Contact

[#] These authors contributed equally.

* Correspondence: YC (cong@sibcb.ac.cn), ZD (dingzhanyu@sibcb.ac.cn), CP (pengchao@sari.ac.cn)

This Supplementary Information PDF includes:

Supplementary Figure 1 – Supplementary Figure 8

Supplementary Table 1 – Supplementary Table 5

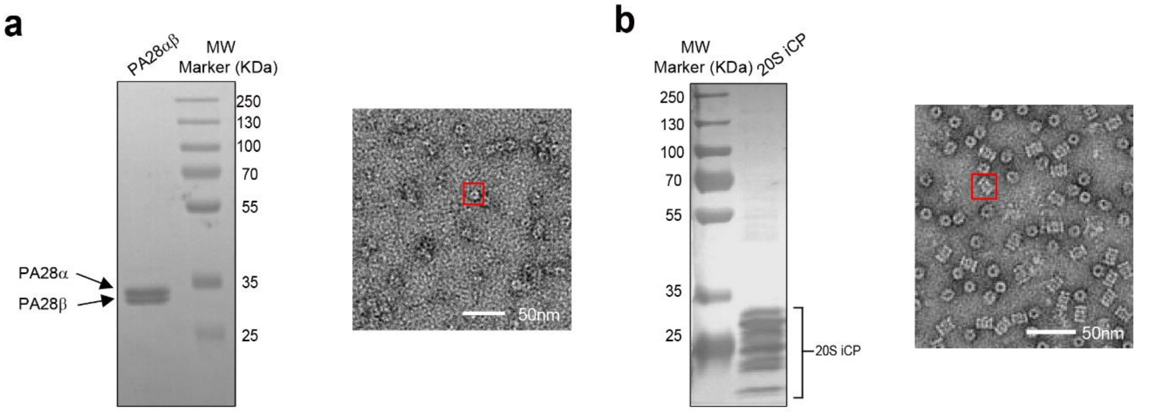

Mass spectra result for PA28αβ

	Coverage	PSMs	Peptides	Unique peptides
PA28α	87.1	273	38	38
PA28β	90.0	218	31	31

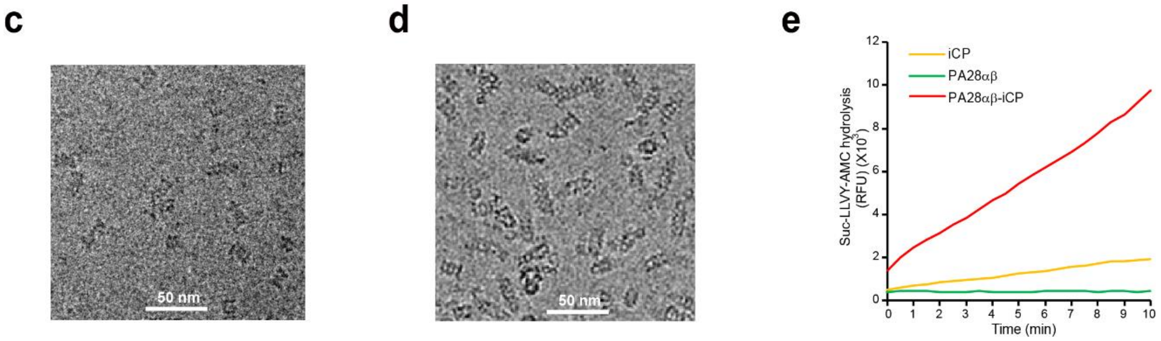

Supplementary Figure 1. Purification and characterization of human PA28αβ and bovine spleen iCP. (a) Characterizations of human PA28αβ using SDS-PAGE, negative-staining electron microscopy (NS-EM), and mass spectrometry analyses. PSMs stands for peptide spectrum matches, which were used to indicate the relative abundance of a certain protein. Unique peptides were used for protein identification. **(b)** Characterizations of bovine spleen iCP using SDS-PAGE and NS-EM. **(c-d)** A representative cryo-EM image (low pass filtered for better visualization) of the *in vitro* reconstituted PA28αβ-iCP proteasome from purified PA28αβ and iCP in the absence of cross-linker (**c**) and presence of cross-linker **(d)**. **(e)** Proteolytic activity assay of the reconstituted PA28αβ-iCP complex against the fluorogenic peptide Suc-LLVY-AMC. RFU, relative fluorescence units. The experiment was repeated three times with the same outcome.

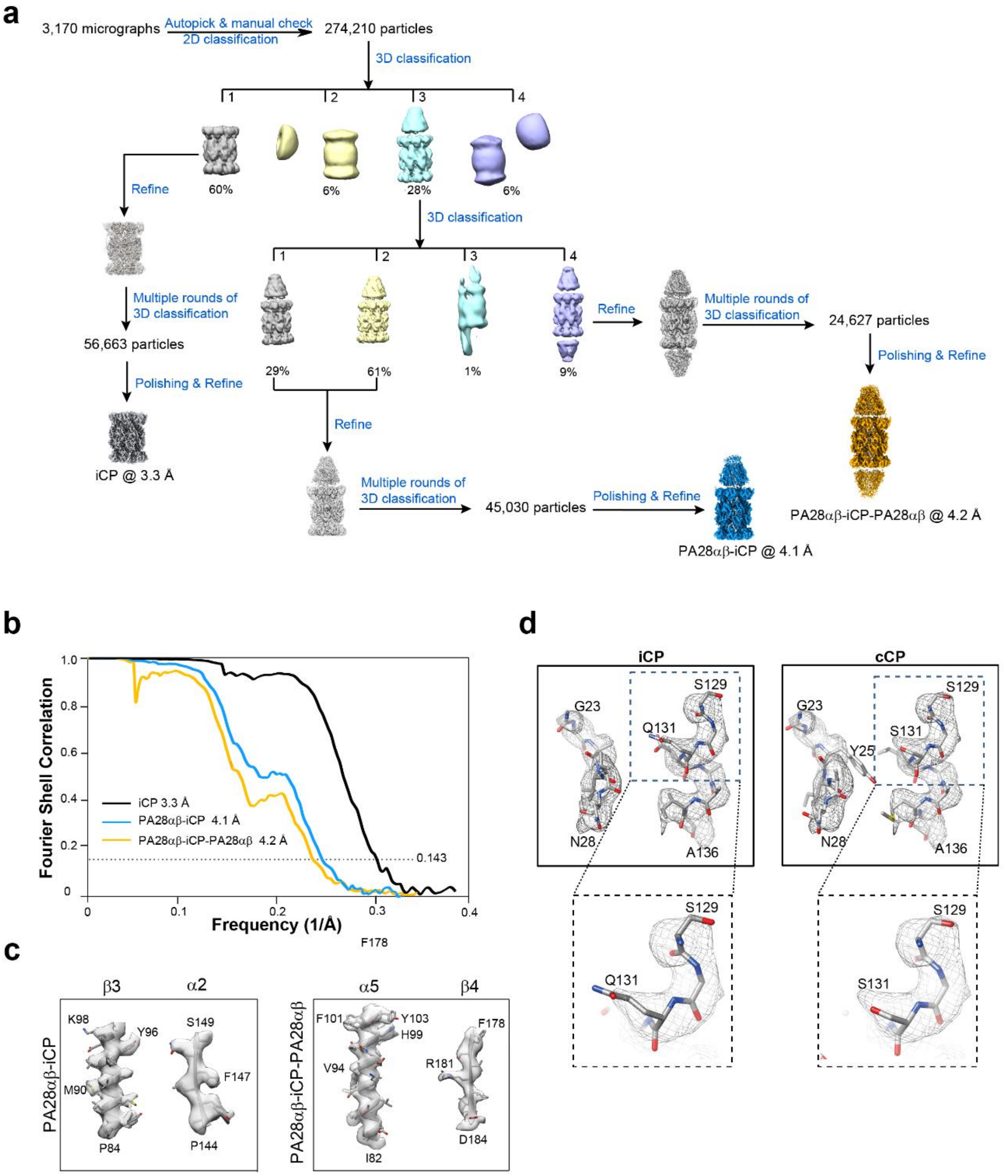

Supplementary Figure 2. Structural analysis of the PA28αβ-iCP proteasome. (a) Work-flow of the PA28αβ-iCP proteasome data processing. More details are available in **Methods**. **(b)** Resolution assessment of our cryo-EM maps using Fourier shell correlation (FSC) at the 0.143 criterion. **(c)** Model-map fitting for PA28αβ-iCP and PA28αβ-iCP-PA28αβ complexes in the iCP portion. **(d)** The zoomed in views in the second row shows that for the same CP density, when cCP β2 S131 is fitted in, there appears to have extra

density (right panel); while this extra density can be partially filled up by iCP Q131 (left panel), implying the partial contribution of the S131 from cCP in this site in addition to that of Q131 from iCP. Still, Q131 of iCP is the better fit to this density possibly due to the dominate population of iCP particles. Also, in the first row, the Y25 in cCP (right panel) is completely out of the density.

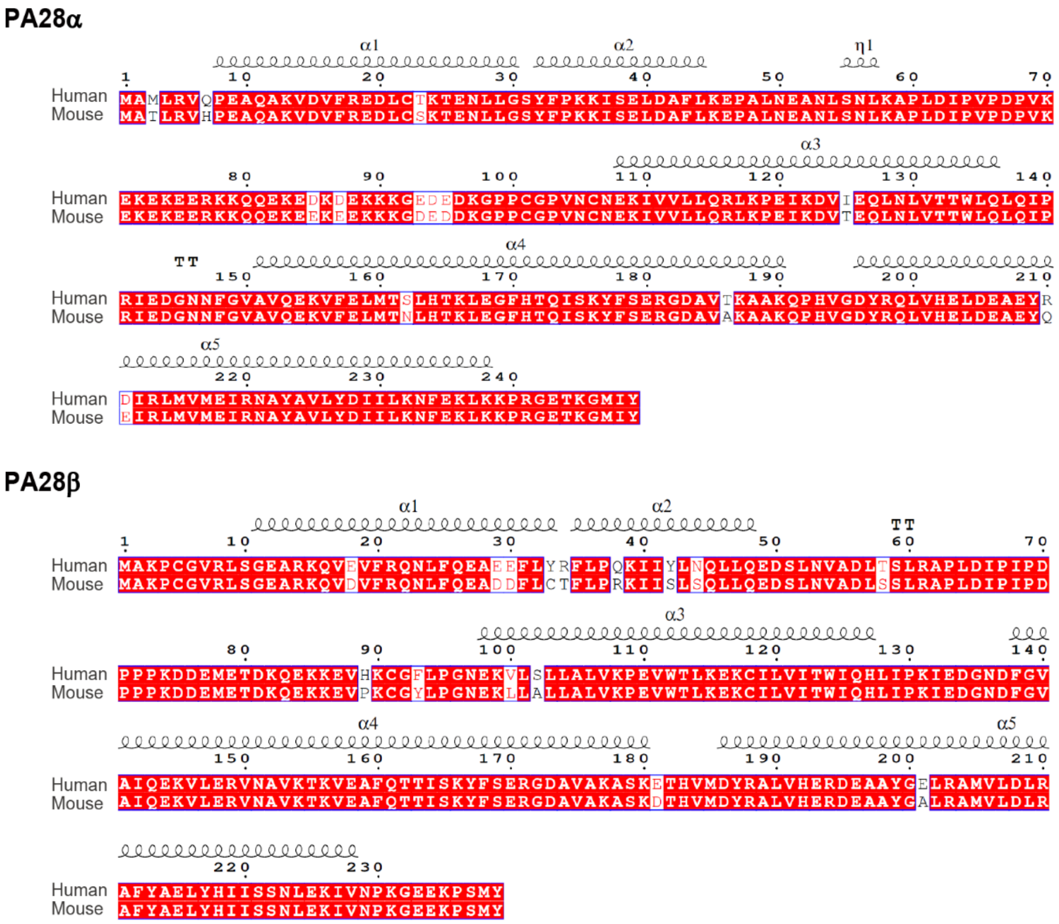

Supplementary Figure 3. High sequence identity between human and mouse PA28αβ. Sequence alignments of human and mouse PA28α (top) and of human and mouse PA28β (bottom), generated by Espript. Showing sequence identities of 94.78% for PA28α between human and mouse, and 93.72% for PA28β between human and mouse.

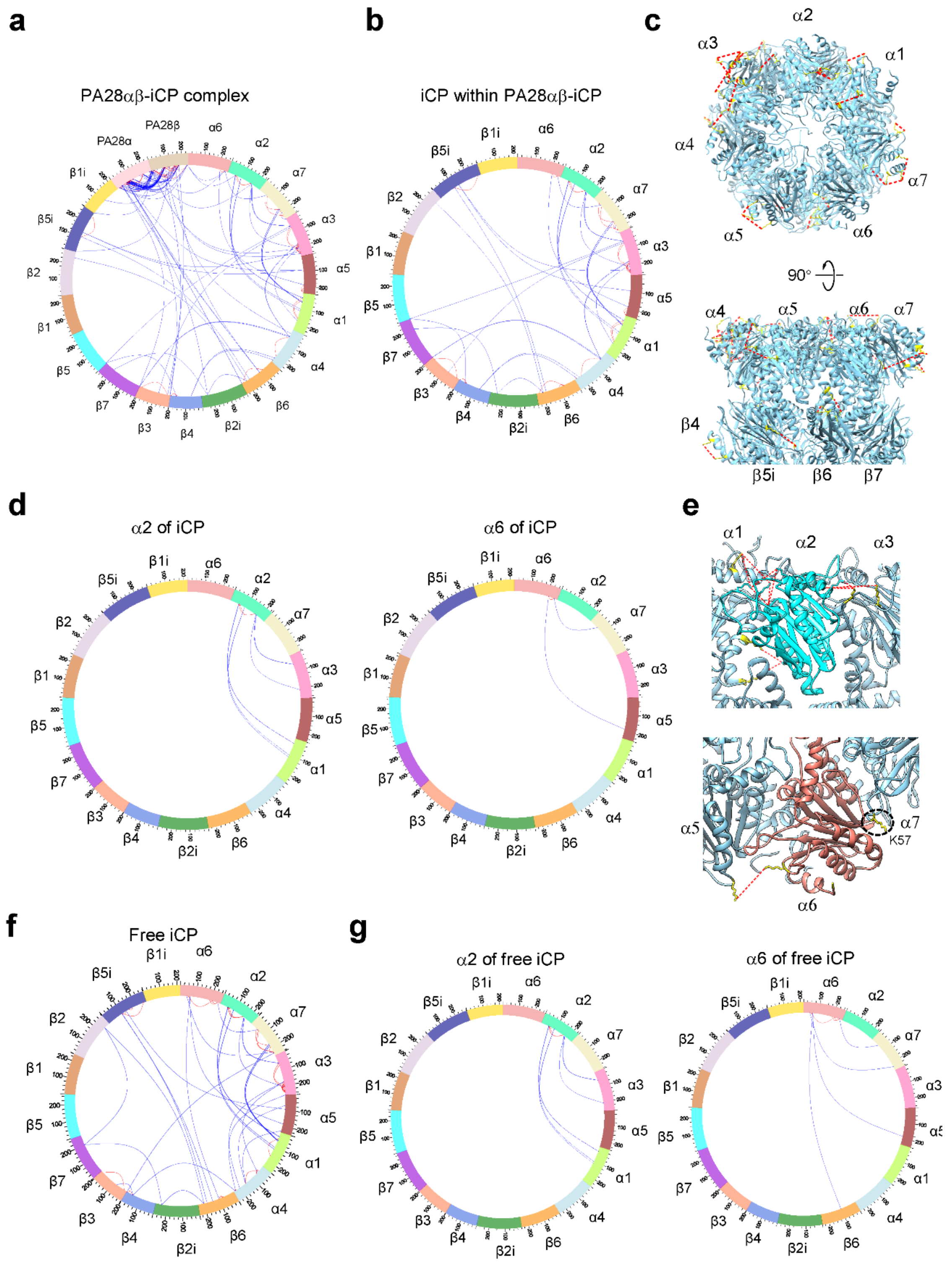

Supplementary Figure 4. Results of XL-MS analysis of PA28αβ-iCP proteasome complex shown by circular plots. (a) Circular plot of all cross-links of PA28αβ-iCP complex detected by XL-MS analysis, with intra-subunit XLs shown in red, and inter-

subunit XLs in blue. We only show the spectra data with E-value below 1.00E-02 in the circular plot, which is followed throughout. **(b)** XLs of the iCP portion from the XL-MS results of the PA28αβ-iCP complex. **(c)** Plot with detected intra subunit cross-links mapped on the core structure. **(d)** We take α2 and α6 core subunits from PA28αβ-iCP complex as examples to illustrate the detected XLs between the subunits of the core proteasome. **(e)** Mapping of those detected intra- and inter-subunit XLs for α2 and α6 subunits on the core particle structure. Due to the dynamic nature of the C-terminus, the α6 C-terminal residue K243 was not resolved in our structure, thus we could not display the XL of α6(K243)- α7(K57) in the model; still the α7(K57) (indicated by dotted black ellipsoid) is fairly close to α6 subunit. **(f)** Circular plot of detected XLs from the free iCP by XL-MS analysis. **(g)** Detected XLs for α2 (left) and α6 (right) from the free iCP XL-MS data, which overall match the XLs detected in the PA28αβ-iCP complex with slightly increased number of detected XLs that might be related to the increased crosslinker concentration. This further validate the reliability of our XL-MS results.

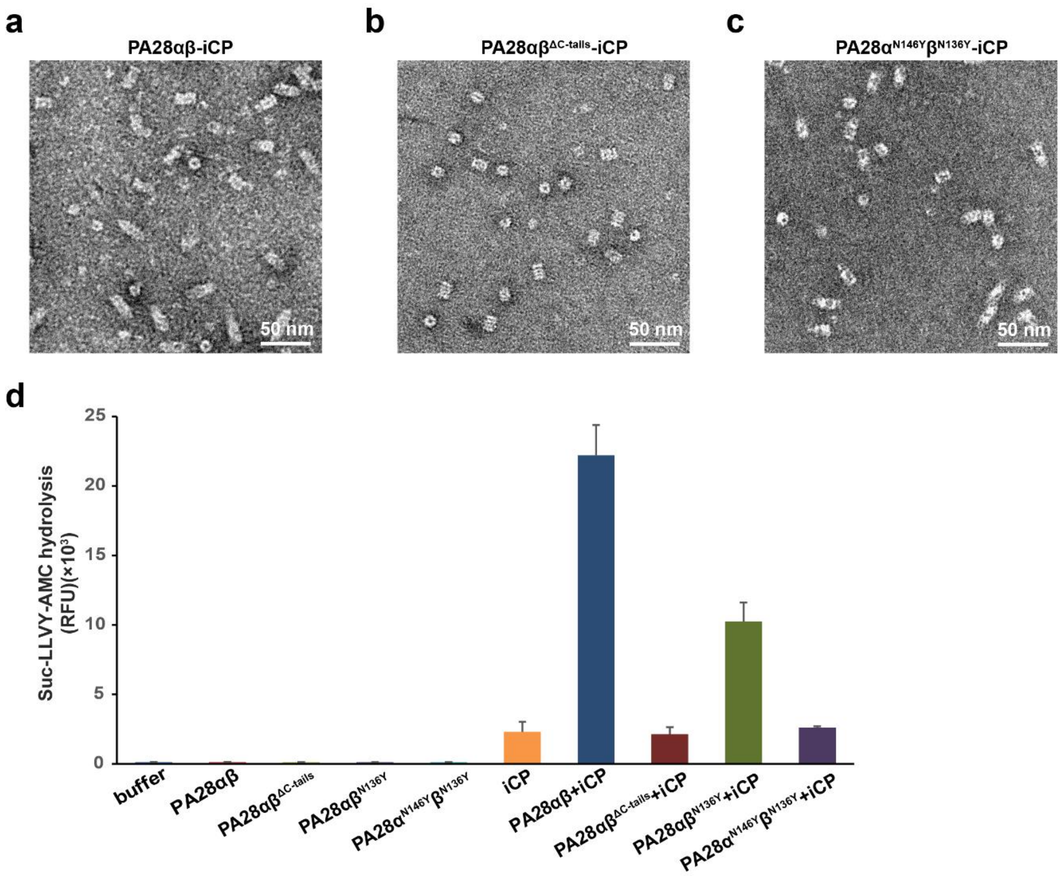

Supplementary Figure 5. Binding assay (through NS-EM analysis) and proteolytic activity assay of wild type PA28αβ or mutated PA28αβ in complex with iCP. (a) Representative NS-EM image of iCP incubated with PA28αβ. **(b)** Similar analyses as in **(a)** but for iCP incubated with the C-terminal truncated PA28αβ$^{ΔC\text{-tails}}$ (truncated K245-Y249 for PA28α and E234-Y239 for PA28β). **(c)** Similar analyses as in **(a)** but for iCP incubated with the activation loop mutated PA28α^{N146Y}β^{N136Y}. **(d)** Proteolytic activity assay of the iCP with PA28αβ, PA28αβ$^{ΔC\text{-tails}}$, PA28α^{N146Y}β^{N136Y}, and PA28αβ^{N136Y} against the fluorogenic peptide Suc-LLVY-AMC. RFU, relative fluorescence units. The experiment was repeated three times with the same outcome.

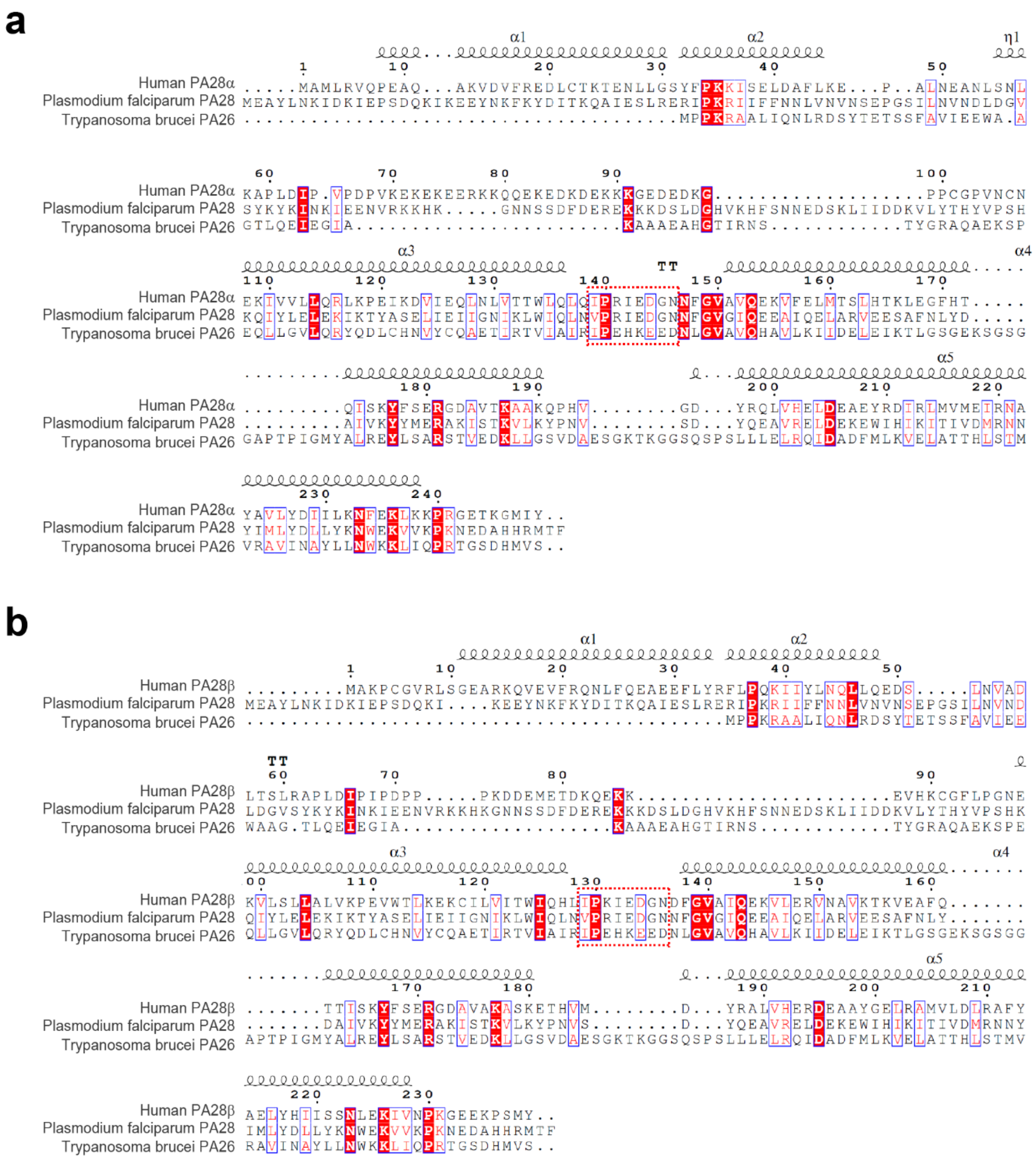

Supplementary Figure 6. Sequence alignments of human, *Plasmodium falciparum* and *Trypanosoma brucei* 11S activator protein chains. (a) Espript representation of sequence alignment of human PA28α, *Pf*PA28 and *Tb*PA26, showing sequence identities of 26.76% between human PA28α and *Pf*PA28, and 15.64% between human PA28α and *Tb*PA26. Here the position of the activation loop was indicated by dotted red frames. This style was followed. **(b)** Espript representation of sequence alignment of human PA28β, *Pf*PA28 and *Tb*PA26, showing sequence identities of 24.38% between human PA28β and *Pf*PA28, and 12.46% between human PA28β and *Tb*PA26.

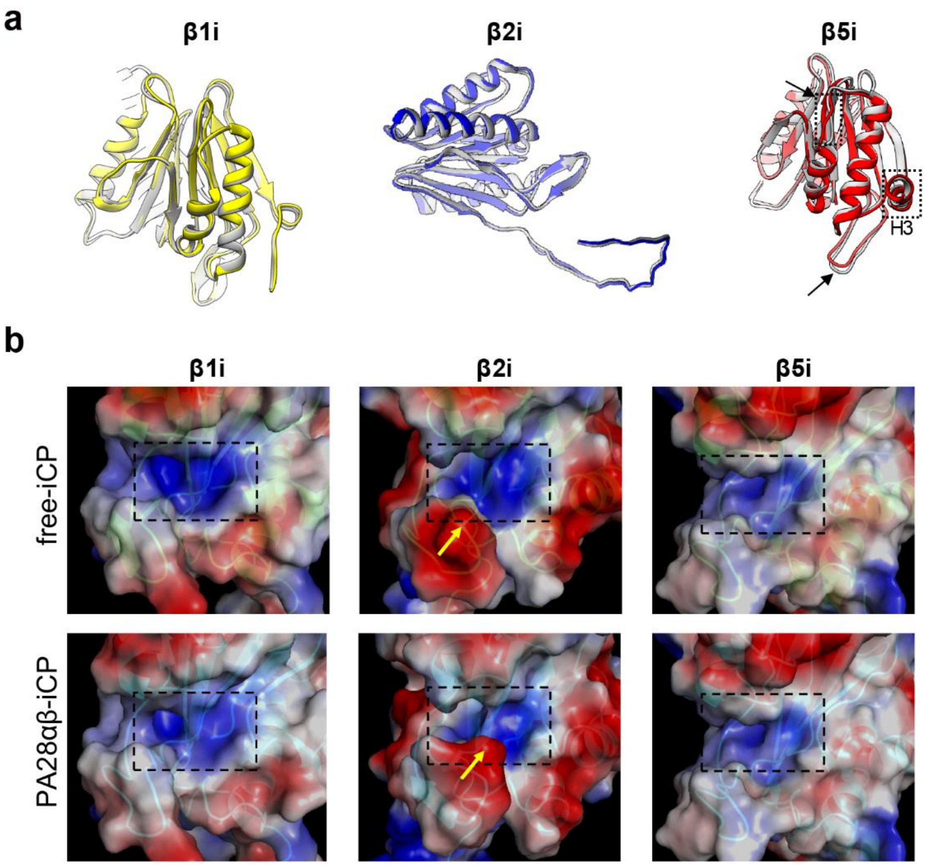

Supplementary Figure 7. Variations of conformation and electrostatic surface property of the three catalytic subunits of bovine iCP induced by PA28αβ binding. (a) Structural superpositions of the three catalytic subunits of our PA28αβ-bound iCP (with β1i, β2i, and β5i in color) on the corresponding subunits of our bovine free iCP (in gray). The observable conformational changes are indicated by black arrow and dotted frames. The rendering style is followed throughout. **(b)** Comparison of electrostatic surface property of β1i, β2i, and β5i between free bovine iCP (top row) and PA28αβ-iCP (bottom row). The proteolytic site location is indicated by black dashed line. In β2i, the surrounding negatively charged loop (indicated by yellow arrow) shows inward movement toward the proteolytic site after binding the PA28αβ.

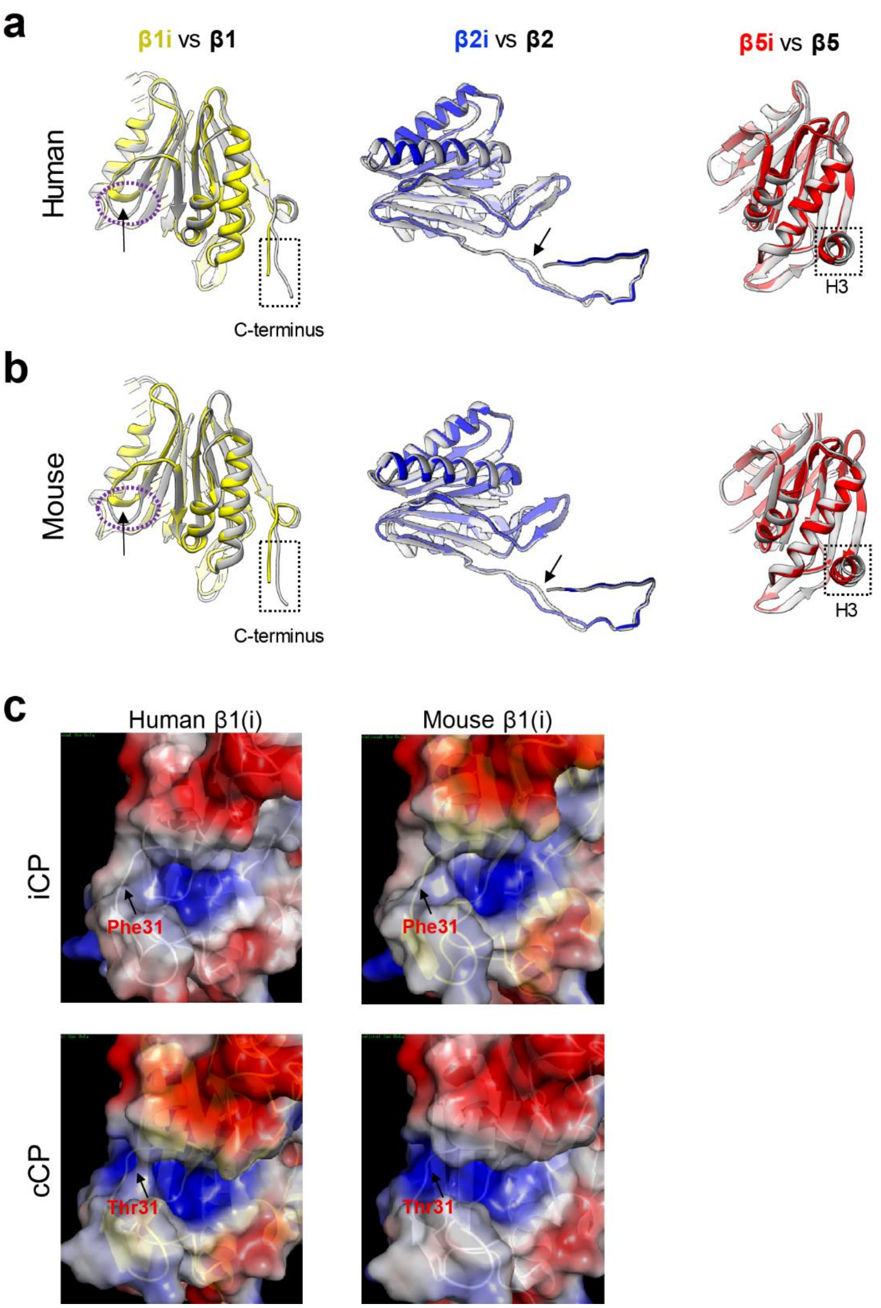

Supplementary Figure 8. Structural comparison and electrostatic surface property comparison of the catalytic subunits between iCP and cCP. (a-b) Superpositions of the structures of **(a)** human iCP (PDB:6AVO) versus human cCP (PDB: 4R3O), and **(b)** mouse iCP (PDB: 3UNH) versus mouse cCP (PDB: 3UNE). **(c)** Electrostatic surface property representations of human β1i versus human β1, and mouse β1i versus mouse β1. With the most distinct residues between β1i and β1 in this region indicated.

Supplementary Table 1. Mass spectrometry analysis of the full composition of the free 20S proteasome from bovine spleen.

Bovine spleen 20S	Coverage	PSMs	Peptides	Unique peptides
PSMB9 /β1i	76	377	12	12
PSMB10 /β2i	42	252	8	8
PSMB8 /β5i	60	215	14	14
PSMB6 /β1	70	72	11	11
PSMB7 /β2	64	74	15	15
PSMB5 /β5	68	74	17	17
PSMB3 /β3	53	242	14	14
PSMB2 /β4	94	390	24	23
PSMB1 /β6	73	304	19	19
PSMB4 /β7	50	305	9	9
PSMA6 /α1	74	497	20	20
PSMA2 /α2	68	318	15	15
PSMA4 /α3	77	419	18	18
PSMA7 /α4	70	375	21	21
PSMA5 /α5	66	355	15	15
PSMA1 /α6	90	324	25	25
PSMA3 /α7	44	181	14	14

Supplementary Table 2. Statistics of cryo-EM data collection, processing, and model validation.

	iCP (EMDB-30825) (PDB 7DR7)	PA28αβ-iCP (EMDB-30824) (PDB 7DR6)	PA28αβ-iCP-PA28αβ (EMDB-30828) (PDB 7DRW)
**Data collection and processing**			
Magnification	18,000x	18,000x	18,000x
Voltage (kV)	300	300	300
Electron exposure ($e^{-}/Å^2$)	38	38	38
Defocus range (μm)	-1.5 ~ -2.8	-1.5 ~ -2.8	-1.5 ~ -2.8
Pixel size (Å)	1.32	1.32	1.32
Symmetry imposed	C2	C1	C1
Initial particle images (no.)	274,210	274,210	274,210
Final particle images (no.)	56,663	45,030	24,627
Map resolution (Å)	3.3	4.1	4.2
FSC threshold	0.143	0.143	0.143
Map resolution range (Å)	2.9-4.4	3.5-6.5	3.5-6.5
**Refinement**			
Initial model used (PDB code)	1IRU	1IRU, 5MX5	1IRU, 5MX5
Model resolution (Å)	3.3	4.1	4.3
FSC threshold	0.143	0.143	0.143
Model resolution range (Å)	3.0-3.6	4.0-6.0	4.0-7.4
Map sharpening *B* factor ($Å^2$)	-65	-96	-82
Model composition			
Non-hydrogen atoms	48,321	59,979	71,673
Protein residues	6,220	8,169	9,108
Ligands			
B factors ($Å^2$)			
Protein	48.73	97.65	47.87
Ligand			
R.m.s. deviations			
Bond lengths (Å)	0.0045	0.0038	0.0035
Bond angles (°)	0.95	0.89	0.92
Validation			
MolProbity score	1.78	1.86	1.61
Clashscore	9.68	9.63	7.91
Poor rotamers (%)	0.35	0.05	0.00
Ramachandran plot			
Favored (%)	96.07	94.91	96.92
Allowed (%)	3.78	4.94	2.97
Disallowed (%)	0.15	0.15	0.11

Supplementary Table 3. Results of XL-MS analysis of PA28αβ-iCP proteasome complex*

Protein1(site)-Protein2(site2) Inter-Proteins	Peptides	E-value	Spec count
α3(210)-PA28α(245)	LSAEKVEIATLTR(5)-GETKGMIY(4)	1.40E-05	13
α6(243)-PA28β(235)	KAQPTQPADEPAEK(1)-GEEKPSMYL(4)	2.39E-05	2
α2(171)-PA28α(245)	NYVNGKTFLEK(6)-GETKGMIY(4)	6.95E-05	15
α2(53)-PA28β(235)	QKSILYDER(2)-GEEKPSMYL(4)	2.18E-04	2
α6(243)-PA28β(15)	KAQPTQPADEPAEK(1)-KQVEVFR(1)	1.93E-08	1
α7(57)- α6(243)	LVLSKLYEEGSNKR(5)-KAQPTQPADEPAEK(1)	7.08E-11	5
α1(30)- α2(53)	LYQVEYAFKAINQGGLTSVAVR(9)-QKSILYDER(2)	5.49E-17	75
α2(92)- α1(104)	KLAQQYYLVYQEPIPTAQLVQR(1)-YKYGYEIPVDMLCK(2)	1.49E-31	42
α3(176)- α4(52)	ATCIGNNSAAAVSMLKQDYK(16)-SVAKLQDER(4)	4.28E-22	21
β5i(133)- α5(91)	LLAKECR(4)-TLIDKAR(5)	7.96E-31	45
β6(204)- β2i(233)	LVKDVFISAAER(3)-ALSSPTKPIER(7)	2.67E-20	63
β7(201)- β2i(233)	EVLEKQPVLSQTEAR(5)-ALSSPTKPIER(7)	5.00E-07	11
β6(204)- β2(237)	LVKDVFISAAER(3)-LDFLRPYSVPNKK(12)	8.48E-11	8
PA28β(180)-PA28α(190)	ASKETHVMDYR(3)-AAKQPHVGDYR(3)	2.64E-21	168
PA28β(180)-PA28α(187)	ASKETHVMDYR(3)-GDAVTKAAK(6)	3.48E-11	109
PA28α(190)-PA28β(177)	AAKQPHVGDYR(3)-GDAVAKASK(6)	1.03E-13	75
…	…	…	…

* We used E-value (1.00E-02) as the threshold to remove extra XL-MS data with lower confidence. For the detected cross-links within iCP or PA28αβ units, due to limited space of the table, here we listed representative results.

Supplementary Table 4. Occupancy status of the 20S pockets of PA28αβ, *Pf*PA28 and *Tb*PA26.

20S pockets	PA28αβ	*Pf*PA28	*Tb*PA26
α1/ α2	++	-	-
α2/ α3	++	-	++
α3/ α4	++	-	++
α4/ α5	++	-	++
α5/ α6	+	-	++
α6/ α7	-	-	-
α7/ α1	-	+	-

++: strong density, +: weak density; -: no density

Supplementary Table 5. Stabilization status of activation loops of PA28αβ, *Pf*PA28, and *Tb*PA26.

20S subunits	PA28αβ	*Pf*PA28	*Tb*PA26
α1	++	+	++
α2	+	-	++
α3	++	-	++
α4	++	-	++
α5	+	+	++
α6	++	++	++
α7	+	++	++

++: well resolved, +: less well resolved; -: not resolved.
